# Water-Soluble, Alanine-Modified Fullerene C_60_ Promotes the Proliferation and Neuronal Differentiation of Neural Stem Cells

**DOI:** 10.3390/ijms23105714

**Published:** 2022-05-20

**Authors:** Haiyuan Ren, Jinrui Li, Ai Peng, Ting Liu, Mengjun Chen, Hongguang Li, Xiaojing Wang

**Affiliations:** 1 Department of Cell Biology, School of Basic Medical Sciences, Cheeloo College of Medicine, Shandong University, Jinan 250012, China; ren15993562826@163.com (H.R.); pengai1007@163.com (A.P.); liuting@sdu.edu.cn (T.L.); 2 Key Laboratory of Colloid and Interface Chemistry, Ministry of Education, School of Chemistry and Chemical Engineering, Shandong University, Jinan 250100, China; ljr@mail.sdu.edu.cn; 3 School of Qilu Transportation, Shandong University, Jinan 250002, China; mjun@sdu.edu.cn

**Keywords:** fullerene C_60_ derivatives, NSCs, proliferation, differentiation, H_2_O_2_

## Abstract

As carbon-based nanomaterials, water-soluble C_60_ derivatives have potential applications in various fields of biomedicine. In this study, a water-soluble fullerene C_60_ derivative bearing alanine residues (Ala-C_60_) was synthesized. The effects of Ala-C_60_ on neural stem cells (NSCs) as seed cells were explored. Ala-C_60_ can promote the proliferation of NSCs, induce NSCs to differentiate into neurons, and inhibit the migration of NSCs. Most importantly, the Ala-C_60_ can significantly increase the cell viability of NSCs treated with hydrogen peroxide (H_2_O_2_). The glutathioneperoxidase (GSH-Px) and superoxide dismutase (SOD) activities and glutathione (GSH) content increased significantly in NSCs treated even by 20 μM Ala-C_60_. These findings strongly indicate that Ala-C_60_ has high potential to be applied as a scaffold with NSCs for regeneration in nerve tissue engineering for diseases related to the nervous system.

## 1. Introduction

Neural stem cells (NSCs) have the potential of self-renewal and multipotent differentiation, which can differentiate into neurons, astrocytes, and oligodendrocytes in the nervous system [1,2]. Because NSCs offer a potential source for cell/tissue replacement therapy, they are ideal candidates as seed cells for neural tissue engineering and regenerative medicine.

Many studies have suggested that the physical and chemical properties of scaffold materials, such as inorganic nanomaterials, have a critical effect on the self-renewal and neuronal differentiation of NSCs in neural tissue engineering [3]. For example, mesoporous silica nanoparticles have been found to be capable of loading neural growth factors to promote neural-cell and neurite growth, even to control the fate of NSCs [4] and help imaging of NSCs distribution in glioma xenografts [5]. Reduced graphene oxide (rGO)/TiO_2_ heterojunction film can be used as a biocompatible flash photo stimulator for effective differentiation of hNSCs into neurons [6]. A nanostructured rGO microfiber was found to support NSCs growth and regulate NSCs differentiation into Tuj1-positive neurons while inhibiting the glial differentiation [7]. Graphene substrates were shown to promote the differentiation of human neural stem cells into neurons [8]. A dense TiO_2_ ceramic with a flat surface can regulate NSCs into Tuj1-positive neural differentiation, while nanoporous TiO_2_ ceramics can induce the glial differentiation of NSCs. These data proved that localized and committed differentiation can be achieved in NSCs culture system by integrating materials with different topographies [9]. Carbon nanotube (CNTs) promoted neurite elongation and the differentiation of NSCs into mature neuronal cells [10]. 

Among the carbon-based nanomaterials, fullerene C_60_ (refers to C_60_ hereafter), with a stable cage-like structure resistant to the actions of both acids and bases, is a promising material in various fields of biomedicine [11,12,13,14]. However, C_60_ is a fully hydrophobic molecule and has poor solubility in water, which greatly limits its biomedical functions. To solve this problem, numerous preparation techniques have been developed, which resulted in a wide range of C_60_ derivatives. Through extensive modification on the surface, water-dispersible C_60_ derivatives could be obtained, opening the door for their utilization in biomedical fields including anticancer/antimicrobial therapy, controlled drug delivery, photodynamic therapy, and cytoprotection [15,16]. Specifically, in the nervous system, the hydroxylated C_60_, i.e., fullerenols, have shown spectacular antioxidant activity to reduce apoptosis in cortical neurons [17]. Hexa (sulfobutyl) C_60_ derivative and trimesic acid (TMA) C_60_ derivative also demonstrated their ability to trap free radicals in the treatment of neurodegenerative diseases [18,19]. Fullerenols and malonic-acid-modified C_60_ can reduce neuronal degeneration related to reactive oxygen species (ROS) by reacting with hydroxyl and superoxide free radicals [20]. Many studies showed the neuroprotective effect of C_60_ derivatives as radical scavengers in in vitro and in vivo stroke models [21,22,23,24]. C_60_ derivatives were found to affect the proliferation and differentiation of NSCs. Aligned C_60_ nanowhiskers (NWs) can orient NSCs and have a high capacity to differentiate into mature neurons. The aligned C_60_ NWs can serve as a functional scaffold for neural tissue engineering [25]. C_60_ and CNTs were found to enhance the proliferation and differentiation of NSCs [26]. Water-soluble C_60_ derivatives with different types of linkages between the C_60_ cage and the solubilizing groups could exert different effects on neural stem cells and glioblastoma cells. The compound bearing residues of phenylbutiryc acids significantly increased the proliferation/survival of NSCs and induced neural repair, while the compound with phenylalanine appendages significantly inhibited the glioblastoma growth [27]. For neural tissue engineering, new water-soluble C_60_ derivatives should be designed, and their effects on NSCs need to be investigated deeply.

In this study, we successfully assembled a water-soluble C_60_ derivative modified with alanine (refers to Ala-C_60_ hereafter). NSCs were used as seed cells to determine whether the Ala-C_60_ can affect the proliferation, migration, and differentiation of NSCs. The results demonstrate that the Ala-C_60_ not only promotes proliferation but also enhances neuronal differentiation under neural differentiation conditions for seven, while inhibiting the migration of NSCs. Most importantly, the Ala-C_60_ can significantly increase the cell viability of NSCs treated with H_2_O_2_. GSH-Px and SOD activities and GSH content increased significantly in NSCs treated even by 20 μM Ala-C_60_. This result proves the anti-oxidative stress effect of the Ala-C_60_. This finding provides the possibility to utilize the Ala-C_60_ as a scaffold with NSCs in neural tissue engineering.

## 2. Results

### 2.1. Preparation and Structure of Ala-C_60_

Ala-C_60_ was synthesized by referring to the literature [28] with modifications. The detailed preparation process can be found in the experimental section. Compared to the previous method, we employed *o*-xylene as a solvent for C_60_ instead of toluene, which improved the yield of the final product due to the much higher concentration of C_60_ in *o*-xylene than in toluene. The employment of dialysis during the purification process gives a complete removal of the inorganic salts and small organic molecules, making the final product highly pure.

**Figure 1:**
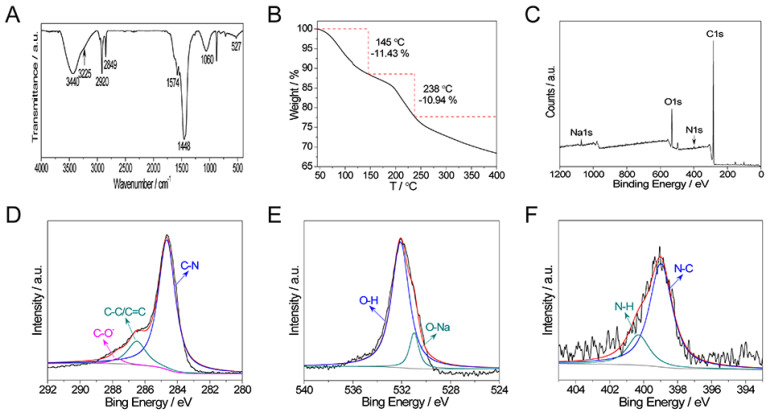
Analyses of the structure and composition of Ala-C_60_. (**A**) FTIR spectrum. (**B**) TGA curve. (**C**) XPS survey. (**D**–**F**) High-resolution XPS spectra of C1s, O1s, and N1s.

The types of the organic functional groups on the surfaces of the Ala-C_60_ were probed by FTIR measurement (Figure 1A). The vibration of N-H (νN-H) and C-N (νC-N) located at 3440, 1060 cm^−1^ reveals the presence of amino groups on the molecules. The peaks at 2920 and 2849 cm^−1^ indicate the presence of alkyl chains from residual tetrabutylammonium cations (TBA^+^). The peaks at 1574 and 1448 cm^−1^ are from COO^−^, and the peak at 575 cm^−1^ is the characteristic absorption of C_60_. A three-step weight loss was confirmed (Figure 1B) from thermogravimetric analysis (TGA) performed under nitrogen. The first weight loss that occurred before 145 °C (11.43 wt%) is assigned to the loss of secondary bound water. The second weight loss before 238 °C (10.94 wt%) can be attributed to the dehydration of the hydroxy groups. The last one above 379 °C originated from the sublimation of C_60_.

X-ray photoelectron spectroscopy (XPS) measurements were used to analyze the composition of Ala-C_60_ (Figure 1C–F and Figure S1). In addition to C1s, O1s, and N1s, the presence of Na1s was also confirmed (Figure 1C and Figure S1). High-resolution C1s spectra (Figure 1D) showed peaks at 284.6, 286.5, and 287.7 eV, which can be assigned to C-N, C-C/C=C, and C-O, respectively, further confirming the connection of amino group and C_60_. The atomic percentages of C, O, N, Na were determined to be 73.3%, 23.15%, 2.09%, and 2.46%, respectively. The weight ratio of C to H was calculated to be ~16 according to elemental analysis (Table 1).

**Figure 2:**
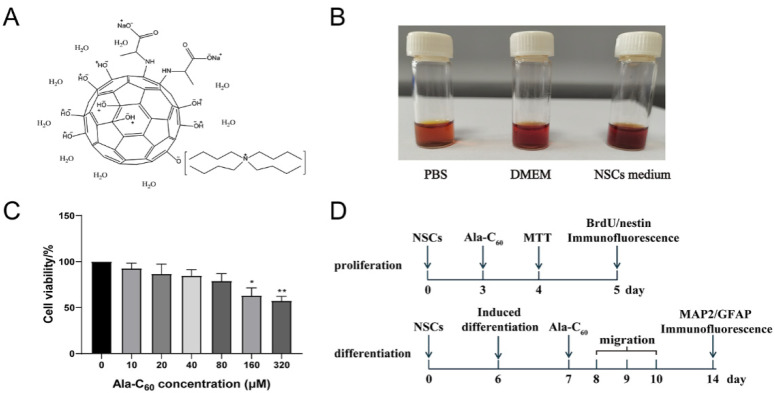
(**A**) Proposed structure of Ala-C_60_. (**B**) Photos of Ala-C_60_ dissolved in PBS (1 mg⋅mL^−1^), DMEM (1 mg⋅mL^−1^), and NSCs medium (1 mg⋅mL^−1^). (**C**) Cell viability of NSCs treated with Ala-C_60_. (**D**) Experimental scheme of Ala-C_60_ on NSCs. Values represent the mean ± SEM, * *p* < 0.05 and ** *p* < 0.01 indicate statistical significance between Ala-C_60_ treatment group and Ala-C_60_ 0 μM group. All statistical according to ANOVA followed by LSD post hoc analysis.

Based the above analyses, the average formula of Ala-C_60_ was calculated to be C_60_(NH-CH(CH_3_)-COO^−^)_2_O^−^_9_H^+^_8_Na^+^_2_TBA^+^⋅9H_2_O (Detailed calculations can be found in supporting information). Its structure is illustrated in Figure 2A.

### 2.2. Cytotoxic Effects of the Ala-C_60_ on NSCs

The solubility of Ala-C_60_ was investigated in detail in phosphate buffer solution (PBS), Dulbecco’s modified eagle medium (DMEM) and NSCs medium. As shown in Figure 2B, Ala-C_60_ can dissolve in these three different mediums to give stable and transparent solutions. Next, the cytocompatibility of the scaffolds was determined using cultured primary NSCs. The primary NSCs derived from Sprague Dawley pregnant rat E13.5 brain. NSCs were cultured as monolayer adherent cells or as suspended neurospheres (Figure S2A,B). The cells expressed nestin, which is a common marker for NSCs (Figure S2C) [29]. NSCs were plated at a density of 1 × 10^4^ cells per well of a 96-well plate with NSCs medium for three days. Cell Counting Kit-8 (CCK) assay was performed after 48 h of incubation with Ala-C_60_ protecting from light. The different concentrations of 10 to 320 μM of Ala-C_60_ were applied in this assay. The concentration of Ala-C_60_ with 160 and 320 μM significantly reduced the cell viability compared to the control group (160 μM: 63.11 ± 16.60%, *p* = 0.024 vs. CTR; 320 μM: 57.52 ± 9.34%, *p* = 0.006 vs. CTR). The cell viability showed no significant difference in groups treated with 10, 20, 40, 80 μM Ala-C_60_ (*p* > 0.05, Figure 2C). This result suggests that Ala-C_60_ had low toxicity on the survival of NSCs even at a relatively high concentration (≥160 μM). Therefore, the concentrations of 20, 40, and 80 μM were set for Ala-C_60_ in the following experiments.

### 2.3. Promotion of the Proliferation and Neuronal Differentiation of NSCs by Ala-C_60_

**Figure 3:**
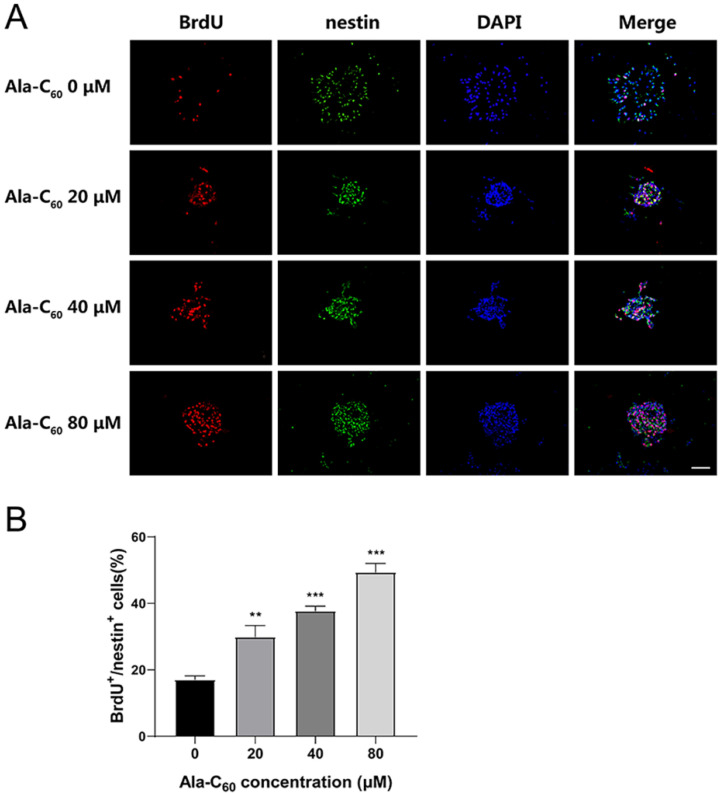
The effect of Ala-C_60_ on the proliferation of cultured NSCs. (**A**) BrdU (red)/nestin (green)-positive cells were identified via immunohistochemical staining. DAPI (blue) was used to label the nuclei. Scale bar = 100 μm. (**B**) Quantitative analysis of BrdU/nestin-positive cells. ** *p* < 0.01, *** *p* < 0.001 according to ANOVA followed by LSD post hoc analysis.

In order to test whether Ala-C_60_ can affect the properties of NSCs, the proliferation and differentiation of NSCs were investigated. The detailed experimental scheme is shown in Figure 2D. First, for the proliferation assay, NSCs were cultured as neurospheres in proliferation medium for three days. Subsequently, NSCs were incubated with 20, 40, and 80 μM Ala-C_60_ for 48 h in the dark. BrdU/nestin double immunofluorescence staining with antibodies against BrdU and nestin was used to label the proliferating NSCs. BrdU, a thymidine analog, can replace thymine (T) into the replicating DNA molecule during cell proliferation [30]. As shown in Figure 3A,B, the proportion of BrdU/nestin-positive cells in the group treated with Ala-C_60_ was significantly higher than that in the control group (17.07 ± 2.77%), and the proportion increase depended on the increase in concentration of Ala-C_60_. The proportions of BrdU/nestin-positive cells treated with 20, 40, and 80 μM Ala-C_60_ were 29.87 ± 6.88%, 37.73 ± 3.51%, and 49.38 ± 5.29%, respectively. This result indicates that Ala-C_60_ can promote the proliferation of NSCs.

**Figure 4:**
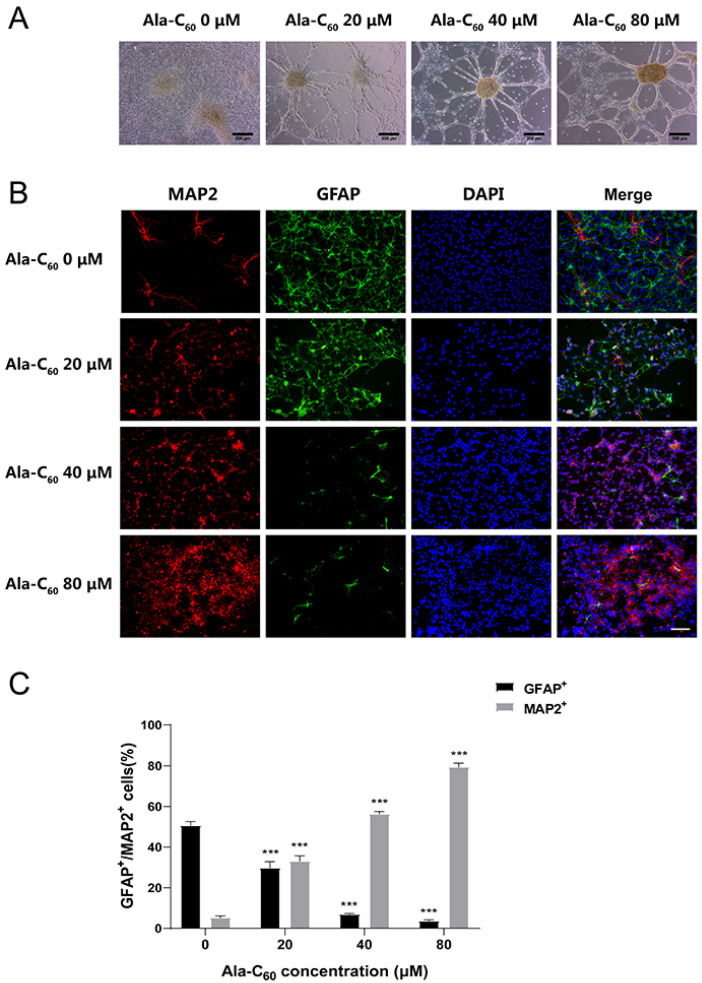
The effect of Ala-C_60_ on the differentiation of cultured NSCs. (**A**) Optical microscopy images of nerve cells on the seventh day of differentiation. Scale bar = 200 μm. (**B**) MAP2 (red)/GFAP (green)-positive cells were identified via immunohistochemical staining. DAPI (blue) was used to label the nuclei. Scale bar = 100 μm. (**C**) Quantitative analysis of MAP2/GFAP-positive cells. *** *p* < 0.001 according to ANOVA followed by LSD post hoc analysis.

Next, the NSCs as neurosphere suspension culture was induced to differentiate by the differentiation medium on the sixth day after plating. Neurospheres and differentiation medium were added to the 24-well plate after treatment with poly-L-lysine. Ala-C_60_ was added into the medium after plating for 24 h. Interestingly, after one week of differentiation, the cells showed a spider web-like morphology compared to those in the control group (Figure 4A). On the seventh day after being treated with Ala-C_60_, double immunofluorescence staining was performed. Microtubule association protein-2 (MAP2), an important component of the cytoskeleton of neurons, is also a specific marker of mature neurons [31]. Meanwhile, glial fibrillary acidic protein (GFAP) is a marker of astrocytes [32]. As shown in Figure 4B,C, with the dose increase of Ala-C_60_, the proportion of MAP2-positive cells in the total number significantly increased, compared with the control group (5.32 ± 1.55%). The percentages of MAP2-positve cells treated with 20, 40, and 80 μM Ala-C_60_ were 33.10 ± 5.18%, 56.30 ± 2.73%, and 79.39 ± 3.90%, respectively. Contrary to the increasing trend of MAP2-positive cells, the percentage of GFAP-positive cells was significantly reduced in the group treated with Ala-C_60_. The percentages of GFAP-positive cells treated with 20, 40, and 80 μM Ala-C_60_ were 29.75 ± 6.22%, 6.97 ± 0.61%, and 3.65 ± 1.00%, compared to the control group (50.63 ± 4.89%, Figure 4C). These results indicated that Ala-C_60_ promoted neuronal differentiation while inhibiting glial differentiation.

### 2.4. Inhibition of the Migration of NSCs by Ala-C_60_

In rodents, NSCs give rise to a large number of neuroblasts that migrate through the rostral migratory stream to the olfactory bulb, where they differentiate into interneurons. Conversely, in the human brain, a large number of neuroblasts migrate into the neocortex instead of the rostral migratory stream during the first few months of life [33]. Hence, migration is a vital process during neurogenesis. 

**Figure 5:**
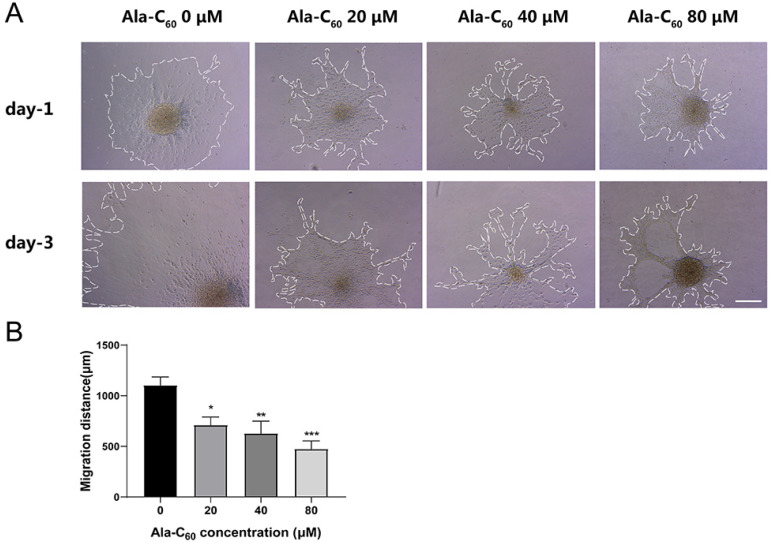
Ala-C_60_ inhibited the migration of cultured neuroblasts. (**A**) Optical microscopy images of the migration distances of neuroblasts on the first and third day. Scale bar =200 μm. (**B**) Quantitative analysis of migration distance. * *p* < 0.05, ** *p* < 0.01, *** *p* < 0.001, according to ANOVA followed by LSD post hoc analysis.

To explore whether Ala-C_60_ affected the migration of NSCs, the migration distance between NSCs progeny and neurospheres was measured during differentiation. After being treated with Ala-C_60_ for 24 and 72 h, we measured the longest diameters of migrating differentiated cells to the neurospheres’ core in each group. The migration distance was 1104.39 ± 278.58 μm in the control group, while the migration distances in groups treated with different concentrations of Ala-C_60_ were 711.36 ± 236.80 μm, 628.39 ± 321.08 μm, and 475.56 ± 193.04 μm, respectively (Figure 5A,B). The groups treated with Ala-C_60_ had a significantly shorter migration distances compared to the control group. These data indicate that Ala-C_60_ inhibited the migration of NSCs progeny. Moreover, the migration was inhibited more severely at a higher concentration of Ala-C_60_.

### 2.5. Antioxidant Ability on NSCs of Ala-C_60_ Induced by H_2_O_2_

**Figure 6:**
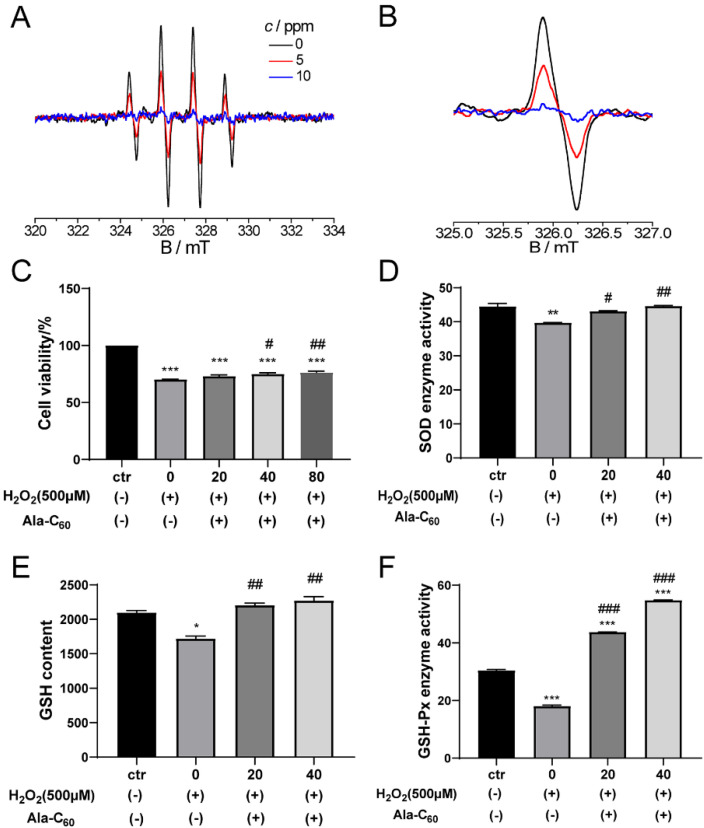
(**A**) ESR spectra of 357 μM H_2_O_2_ aqueous solution in the presence of different concentrations of Ala-C_60_. Each solution contains 7.14 mM DMPO as the probe. (**B**) Magnified spectra in the range of 325.0–327.0 mT (the peak indicated by the ellipse in **A**). (**C**–**F**) Cell viability (**C**), intracellular superoxide dismutase activity (**D**), intracellular glutathione content (**E**), and intracellular glutathione peroxidase (**F**) of cultured NSCs treated with 500 μM H_2_O_2_ for 24 h after addition of Ala-C_60_. * *p* < 0.05, ** *p* < 0.01, *** *p* < 0.001 indicate statistical significance between H_2_O_2_ treatment group and CTR group. ^#^ *p* < 0.05, ^##^ *p* < 0.01, ^###^ *p* < 0.001 indicate statistical significance between Ala-C_60_ treatment group and Ala-C_60_ 0 μM group. All statistics according to ANOVA followed by LSD post hoc analysis.

H_2_O_2_ is the classical reagent to study oxidative stress. First, the capability of Ala-C_60_ to scavenge the hydroxyl radical (OH·) produced by H_2_O_2_ was investigated by electron spin resonance (ESR) measurements. As shown in Figure 6A,B, the intensity of the peak decreased continuously with the increasing concentration of the Ala-C_60_. The initial quenching of OH^−^ is pretty fast, with a quenching efficiency of up to 49.8% at a concentration as low as 4 μg·mL^−1^ (∼5 ppm). It then slows down at relatively large concentrations of the Ala-C_60_, which reaches ∼89% at 8.6 μg·mL^−1^.

Next, we set up the oxidative stress model, using NSCs treated with H_2_O_2_ to explore the effect of Ala-C_60_ on oxidative stress. In order to choose an optimal concentration of H_2_O_2_ and treatment time, we added various concentrations of H_2_O_2_ (100~2000 μM), respectively, to NSCs (Figure S3A–C). On the second day after neural stem cell seeding, H_2_O_2_ was added to the 96-well plate after poly-L-lysine adherence treatment, and the cell viability was measured at 24 h, 48 h, and 72 h. At 24 h, the concentration of H_2_O_2_ higher than 500 μM significantly reduced the cell viability (500 μM: 74.01 ± 5.91%; 1000 μM: 62.76 ± 0.87%; 2000 μM: 59.99 ± 1.05%; Figure S3A). At 48 h, the cell viability of NSCs treated with H_2_O_2_ (≥500 μM) fell drastically compared to the control group (500 μM: 64.56 ± 2.51%; 1000 μM: 51.67 ± 3.66%; 2000 μM: 41.02 ± 1.60%; Figure S3B). At 72 h, the cell viability of NSCs treated with H_2_O_2_ (≥500 μM) fell lowest compared to the control group (500 μM: 65.81 ± 7.86%; 1000 μM: 37.27 ± 5.49%; 2000 μM: 29.42 ± 0.62%; Figure S3C). Finally, we chose 500 μM H_2_O_2_ to treat NSCs for 24 h to establish an oxidative stress model. Thus, the concentration and time were determined in the following experiments.

In order to test the effect of Ala-C_60_ on NSCs during oxidative stress, different concentrations of Ala-C_60_ (20, 40, 80 μM) were added to NSCs medium treated with 500 μM H_2_O_2_ for 24 h. After 24 h, the cell viability was measured. In Figure 6C, cell viability of NSCs increased as the concentration of Ala-C_60_ increased. Especially, treatment with 40 and 80 μM of Ala-C_60_ significantly increased the cell viability of NSCs with oxidative stress (*p* = 0.023 and *p* = 0.004). This result suggests that Ala-C_60_ can inhibit H_2_O_2_-induced cell damage.

SOD is an important component of the antioxidant enzyme system in organisms [34]. GSH-Px is an important enzyme that catalyzes the decomposition of hydrogen peroxide widely in the body [35]. GSH, which catalyzes H_2_O_2_ into H_2_O, is the most important non-enzymatic antioxidant in the body and an important factor in measuring the body’s antioxidant capacity [36]. These indicators were used to evaluate the antioxidant capacity and repair ability of Ala-C_60_ on the NSCs treated with H_2_O_2_. Figure 6D shows that the activity of the SOD enzyme was significantly reduced when NSCs treated with H_2_O_2_. However, treatment with 20 and 40 μM of Ala-C_60_ for 3 h significantly increased the activity of the SOD enzyme (*p* = 0.03 and *p* = 0.007). Furthermore, oxidative stress can also cause a decrease in the content of GSH and the activity of GSH-Px. After treatment with 20 and 40 μM of Ala-C_60_, the content of GSH almost recovered to the level of the control group, and the activity of GSH-Px was much higher than that of the control group (Figure 6E,F). These results prove that Ala-C_60_ has the antioxidant ability and can also increase the activity of antioxidant enzymes and repair the damage of NSCs induced by H_2_O_2_.

## 3. Discussion

In the present study, we successfully synthesized a water-soluble fullerene C_60_ derivative bearing alanine residues. A high concentration (≥160 μM) of Ala-C_60_ had low toxicity on the survival of NSCs. The effects of Ala-C_60_ on the property of NSCs were investigated. First, Ala-C_60_ promoted the proliferation of NSCs, induced NSCs to differentiate into neurons, and inhibited the migration of NSCs. Most importantly, Ala-C_60_ had a significant neuroprotective effect on the cell damage of NSCs induced by H_2_O_2_. The activity of antioxidant enzymes (GSH-Px and SOD) and the content of GSH increased significantly in NSCs treated with Ala-C_60_. These findings strongly indicate that Ala-C_60_ has high potential to be applied as a scaffold with NSCs for regeneration in nerve tissue engineering for diseases related to the nervous system.

There were active debates about the toxicity of C_60_ and its derivatives in the past [37,38,39]. The toxicity of nine different types of water-soluble C_60_ derivatives was investigated on the NSCs, where the IC_50_ value was found to vary from 200 nM to 1.5 mM [27]. The different toxicity of C_60_ derivatives can depend significantly on the chemical structure of the appended organic addends and on the solubility of the compounds in aqueous media. Our data show that Ala-C_60_ had no significant effect on the cell viability of NSCs at a concentration lower than 160 μM. However, a relatively high concentration (160 μM and 320 μM) of Ala-C_60_ reduced the cell viability. The low toxicity of Ala-C_60_, combining its good solubility in various mediums, makes it suitable for study and application in biomedicine.

Two fundamental properties of NSCs are the ability to self-renew and to give rise to differentiated progeny. The self-renewal of NSCs can be performed by symmetric or asymmetric cell division. Symmetric cell divisions of NSCs generate two daughter cells sharing the same identity, which is also the same as that of the mother cell. In asymmetric self-renewing cell division, the two daughter cells generated from an NSC have different identities, but one of them shares the same identity as that of the mother cell [40]. In addition, NSCs can differentiate into lineage-specific cells, such as neurons, oligodendrocytes, and astrocytes [41]. As seed cells, it is assumed that NSCs are to provide a regenerative source for new neurons in neural tissue engineering and regenerative medicine. The self-renewal and differentiation of NSCs are affected by many intrinsic and extrinsic factors. Many C_60_ derivatives have been shown to affect the proliferation and differentiation of NSCs [25,26,27]. Our data show that Ala-C_60_ not only promotes proliferation but also enhances neuronal differentiation. Nevertheless, at present, how Ala-C_60_ affects proliferation and differentiation remains an open question.

In neural tissue engineering, the differentiated cells derived from NSCs need to migrate to the destination. However, our data show Ala-C_60_ has a negative effect on migration. These should be additional factors that can promote migration to help Ala-C_60_ and NSCs in the application of neural tissue engineering. Like neurotrophic factors, glial cell line-derived neurotrophic factor (GDNF) has a chemoattractant effect in the migration of neuronal precursor cells along the rostral migratory stream [42]. Furthermore, for non-classical neurotrophic factors, mesencephalic astrocyte-derived neurotrophic factor (MANF), together with its homolog cerebral dopamine neurotrophic factor (CDNF), can promote the migration of neural progenitor cells in the stroke model [43,44].

It is essential for the safety of the biological system to balance the oxidation and antioxidant activity of the body. Massive free radical production by normal cellular metabolism, as well as abnormal reactions, cause cell damage. The structure of C_60_ makes it capable of reacting with free radicals, such as superoxide, hydroxyl radicals, and hydrogen peroxide [45]. Two different fullerenes, C_60_ and C_82_, conjugated with three transition metals, were used as an antioxidant [46]. The antioxidant ability of C_60_ has been widely used in cosmetics and skincare products as an antiageing agent [47]. Fullerene derivatives in Alzheimer’s disease had radical scavenging ability, ROS activation, and ability to interact with peptides [45]. Hydrogen peroxide originates from the enzymatic or spontaneous dismutation of superoxide anions, which are the byproducts of a wide and ubiquitous variety of oxidases [48]. H_2_O_2_ has widely been used to induce cellular oxidative stress in different cell lines. Previous studies have revealed that cultured NSCs are sensitive to the oxidative stress induced by H_2_O_2_ [48,49,50,51,52]. In this study, the activity of antioxidant enzymes (GSH-Px and SOD) and the content of GSH in NSCs reduced after treatment with H_2_O_2_. These findings were consistent with the previous studies [48,50]. Furthermore, our results show that Ala-C_60_ has the antioxidant ability. Ala-C_60_ may be a therapeutic candidate combined with NSCs for the treatment of neurological diseases mediated by oxidative stress.

In summary, we have synthesized a water-soluble fullerene C_60_ derivative bearing amino acid (alanine) residues. With careful structural analysis, the average form of this C_60_ derivative (Ala-C_60_) was obtained. The effects of Ala-C_60_ on the proliferation, differentiation, and migration of NSCs were explored in detail. Our findings reveal that Ala-C_60_ can promote the proliferation of NSCs, induce NSCs to differentiate into neurons, and inhibit the differentiation of astrocytes. In addition, it can also significantly increase the antioxidant ability of NSCs after oxidative stress induced by H_2_O_2_. These findings strongly indicate that Ala-C_60_ has high potential to be applied for regeneration in nerve tissue engineering for diseases related to the nervous system. Considering that the family of amino acids is rich and the synthetic methodology of water-soluble C_60_ derivatives could be further optimized, we believe this type of C_60_ derivatives could find a wide range of applications in biomedicine.

## 4. Materials and Methods

### 4.1. Chemicals and Reagents

C_60_ (99.5%) was purchased from the Suzhou Dade Carbon Nanotechnology Co., Ltd. (Suzhou, China). Tetrabutyl ammoniumhydroxide (TBAH; 40 wt% in H_2_O) was purchased from Sigma (St. Louis, MO, USA). Sodium hydroxide (NaOH, AR) and alanine (AR) were bought from the Sinopharm Chemical Reagent Co. (Shanghai, China). Other chemicals, including ethanol, *o*-xylene, and hydrochloric acid (HCl), were obtained from local suppliers with the highest purity. All the chemicals were used as received unless other stated.

### 4.2. Synthesis of Ala-C_60_

The water-soluble C_60_ derivative used in this study, named Ala-C_60,_ was synthesized following the procedures reported in the literature [28] with modifications. Briefly, a stock solution of C_60_ in *o*-xylene was prepared with a concentration of 5 mg·mL^−1^. Then, 0.884 g of alanine was added to 15 mL of NaOH aqueous solution (1 g·mL^−1^). After alanine was totally dissolved, 45 mL of ethanol was added to obtain the stock solution of alanine. To a round-bottom flask containing 90 mL stock solution of C_60_, the stock solution of alanine was added dropwise under stirring, followed by the addition of 10 drops of TBAH (40 wt%). The mixture was allowed to react in the dark at 25 °C until the upper organic phase became colorless. The dark brown bottom phase was separated with a separating funnel, and the upper phase was washed twice with water. Then, the aqueous phase was combined, and its pH value was adjusted to ~7.0 with HCl. After that, the solution was subjected to dialysis against water with a dialysis tube with a molecular cut-off of 100 Da. The conductivity of the exudate was monitored with a conductivity meter, and dialysis was stopped when the conductivity of the exudate was below 10 μS/cm. The solution was taken out and filtered to remove any precipitates. Finally, water was evaporated in an oven at 60 °C to obtain the target product in a crystallized form.

### 4.3. Isolation of NSCs and Cell Cultures

Following previous protocol [53], NSCs were obtained from E13.5 Sprague Dawley rats embryonic cortical neuroepithelium. The tissues were dissected and digested with 0.2% papain (Sigma, St. Louis, MO, USA) at 37 °C for 30 min. Cells were counted and plated in DMEM/F12 medium (Invitrogen, Carlsbad, CA, USA) supplemented with 2% B_27_ (Invitrogen, Carlsbad, CA, USA), 20 ng·mL^−1^ recombinant human EGF, and 20 ng·mL^−1^ recombinant human basic FGF (both purchased from Millipore, Billerica, MA, USA). NSCs were cultured as adherent cells or suspended neurospheres. For the adherent culture, plates were coated with poly-L-lysine before the cell plating. However, for suspended neurosphere culture, no coating was needed. NSCs were passaged approximately every seven days.

### 4.4. Cell Viability Assay

Cell Counting Kit-8 (CCK) assay purchased from Biosharp (Hefei, China) was used to analyze cell viability. The concentrations of Ala-C_60_ were 10, 20, 40, 80, 160, 320 μM. Neural stem cells were plated as density of 1 × 10^4^ cells/well in 96-well plates for three days and then exposed to Ala-C_60_ for 48 h. The original medium was discarded, and 100 μL fresh medium sample was added to each well, together with 10 μL of CCK-8 solution. Absorbance was measured at 450 nm by a microplate reader.

### 4.5. BrdU Incorporation

We investigated the effect of Ala-C_60_ on the proliferative activity of NSCs by bromodeoxyuridine (BrdU, Abcam, Cambridge, MA, USA) incorporation. NSCs were plated onto poly-L-lysine-coated coverslips at a density of 1.5 × 10^4^/well in 24-well plates. After 72 h, Ala-C_60_ with different concentrations (20, 40, and 80 μM) were added to the 24-well plate, respectively, followed by the addition of 5 μL BrdU to each well on the fifth day. Two hours later, the cells were fixed in 4% paraformaldehyde and processed for immunofluorescence staining.

In brief, the cells were incubated in 2 N hydrochloric acid for 30 min and 10% donkey serum (DS) for 30 min at room temperature. Cells were incubated overnight with sheep polyclonal anti-BrdU antibody (1:400; Abcam, Cambridge, MA, USA) and mouse monoclonal anti-nestin antibody (1:400; Millipore, Billerica, MA, USA) in TBS containing 0.4% Triton X-100 at 4 °C. After three washings with PBS, the cells were incubated by the secondary antibody solution containing donkey anti-mouse Alexa-Fluor 488 (1:500; Abcam, Cambridge, MA, USA) and donkey anti-sheep Alexa-Fluor 594 (1:500; Life Technologies, Carlsbad, CA, USA) in TBS-Triton supplemented with DS for 30 min at room temperature. 

### 4.6. Differentiation Assay and Immunocytochemistry Staining

NSCs were allowed to differentiate for seven days in a differentiation medium supplemented with 1% fetal bovine serum (FBS) and without EGF and bFGF. To the medium, Ala-C_60_ with varying concentrations were added. On the seventh day of differentiation, the cells were fixed by 4% paraformaldehyde (PFA) for 10 min at room temperature for immunostaining of neuronal and astrocyte markers. The cells were washed three times with PBS to remove PFA, and then incubated with 10% donkey serum for 30 min at room temperature. The cells were further incubated overnight at 4 °C with primary antibody neuronal marker, rabbit polyclonal anti-microtubule-associated protein 2 (MAP2; 1:200) (Servicebio, Wuhan, China), and glial marker, mouse monoclonal anti-glial fibrillary acidic protein (GFAP; 1:500) (Servicebio, Wuhan, China) in TBST (TBS + 0.4% Triton-X) supplemented with normal donkey serum. After three washings with PBS, the cells were incubated by the secondary antibody solution containing donkey anti-mouse Alexa-Fluor 488 and donkey anti-rabbit Alexa-Fluor 594 (1:500; Abcam, Cambridge, MA, USA) in TBS-Triton supplemented with DS for 30 min at room temperature in the dark. Thereafter, the cells were nuclearly stained by incubating in an anti-fluorescence quencher containing DAPI (1:1000) for 5 min. A fluorescent microscope (Olympus, Allentown, PA, USA) was utilized to capture representative pictures to obtain merging images using ImageJ software. The values of neurons and astrocytes were counted, and the mean of each was calculated as a percentage.

### 4.7. Measurement of SOD, GSH-Px Sctivity and GSH Content

Inoculate NSCs into a 6-well plate for suspension culture. NSCs were treated with H_2_O_2_ (Sigma, 3 wt% in H_2_O, St. Louis, MO, USA) for 24 h to obtain cells damaged by oxidative stress. After treatment with Ala-C_60_ for 3 h, the cells were collected. Cell lysates were prepared with PBS or cell lysis buffer and centrifuged at 4 °C, 12,000 rpm, 20 min. SOD activity, GSH-Px activity and GSH levels were measured separately using a commercially available assay kit (Nanjing Jiancheng Bioengineering Institute, Nanjing, China). The SOD activity and GSH-Px activity, and GSH levels of neural stem cells were calibrated by protein content, and the protein determination was measured by the BCA method (Solarbio, Beijing, China).

### 4.8. Statistical Analysis

Statistical analysis was performed using GraphPad Prism 8. Graphs for each parameter were plotted for the averages obtained, and the error bars represented the SEM of the values. All data presented are means or means ± standard deviations. According to a one-way ANOVA, the variance in different groups was statistically analyzed to evaluate its significance, and *p* < 0.05 were recognized as statistically significant.

## Figures and Tables

**Table 1:** The element analysis of Ala-C_60_.

Weight (mg)	C (%)	H (%)	N (%)
3.0640	50.93	3.505	2.99

## Supplementary Materials

The following supporting information can be downloaded at: https://www.mdpi.com/article/10.3390/ijms23105714/s1.

## Abbreviations

Ala-C_60_	fullerene C_60_ derivative bearing alanine residues
NSCs	neural stem cells
H_2_O_2_	hydrogen peroxide
GSH-Px	glutathioneperoxidase
SOD	superoxide dismutase
GSH	glutathione
CNTs	carbon nanotube
TMA	trimesic acid
ROS	reactive oxygen species
NWs	nanowhiskers
FTIR	Fourier Transform infrared spectroscopy
TBA^+^	tetrabutylammonium cations
TGA	thermogravimetric analysis
XPS X	ray photoelectron spectroscopy
PBS	phosphate buffer solution
DMEM	dulbecco’s modified eagle medium
CCK-8	cell counting kit-8
BrdU	5-Bromodeoxyuridinc
DAPI	4,6-Diamidino-2-phenylindole dihydrochloride hydrate
MAP2	microtubule association protein-2
GFAP	glial fibrillary acidic protein
OH·	hydroxyl radical
ESR	electron spin resonance
DMPO	5,5-Dimethyl-1-pyrroline N-oxide
GDNF glial cell line	derived neurotrophic factor
MANF mesencephalic astrocyte	derived neurotrophic factor
CDNF	cerebral dopamine neurotrophic factor
TBAH	tetrabutyl ammoniumhydroxide
NaOH	sodium hydroxide
HCL	hydrochloric acid
EGF	epidermal growth factor
bFGF	basic fibroblast growth factor
DS	donkey serum
FBS	fetal bovine serum
PFA	paraformaldehyde
BCA	bicinchoninic acid

## References

[1] Davis A.A., Temple S. (1994). A self-renewing multipotential stem cell in embryonic rat cerebral cortex. Nature.

[2] Reynolds B.A., Weiss S. (1992). Generation of neurons and astrocytes from isolated cells of the adult mammalian central nervous system. Science.

[3] Zhang B., Yan W., Zhu Y., Yang W., Le W., Chen B., Zhu R., Cheng L. (2018). Nanomaterials in Neural-Stem-Cell-Mediated Regenerative Medicine: Imaging and Treatment of Neurological Diseases. Adv. Mater..

[4] Solanki A., Shah S., Yin P.T., Lee K.-B. (2013). Nanotopography-mediated reverse uptake for siRNA delivery into neural stem cells to enhance neuronal differentiation. Sci. Rep..

[5] Cheng S.-H., Yu D., Tsai H.-M., Morshed R.A., Kanojia D., Lo L.-W., Leoni L., Govind Y., Zhang L., Aboody K.S. (2016). Dynamic In Vivo SPECT Imaging of Neural Stem Cells Functionalized with Radiolabeled Nanoparticles for Tracking of Glioblastoma. J. Nucl. Med..

[6] Akhavan O., Ghaderi E. (2013). Flash photo stimulation of human neural stem cells on graphene/TiO_2_ heterojunction for differentiation into neurons. Nanoscale.

[7] Guo W., Qiu J., Liu J., Liu H. (2017). Graphene microfiber as a scaffold for regulation of neural stem cells differentiation. Sci. Rep..

[8] Park S.Y., Park J., Sim S.H., Sung M.G., Kim K.S., Hong B.H., Hong S. (2011). Enhanced Differentiation of Human Neural Stem Cells into Neurons on Graphene. Adv. Mater..

[9] Mou X., Wang S., Guo W., Ji S., Qiu J., Li D., Zhang X., Zhou J., Tang W., Wang C. (2016). Localized committed differentiation of neural stem cells based on the topographical regulation effects of TiO_2_ nanostructured ceramics. Nanoscale.

[10] Huang Y.-J., Wu H.-C., Tai N.-H., Wang T.-W. (2012). Carbon Nanotube Rope with Electrical Stimulation Promotes the Differentiation and Maturity of Neural Stem Cells. Small.

[11] Azizi-Lalabadi M., Hashemi H., Feng J., Jafari S.M. (2020). Carbon nanomaterials against pathogens; the antimicrobial activity of carbon nanotubes, graphene/graphene oxide, fullerenes, and their nanocomposites. Adv. Colloid Interface Sci..

[12] Kraevaya O.A., Novikov A.V., Shestakov A.F., Ershova E.S., Savinova E.A., Kameneva L.V., Veiko N.N., Schols D., Balzarini J., Kostyuk S.V. (2020). Water-soluble fullerene-based nanostructures with promising antiviral and myogenic activity. Chem. Commun..

[13] Meshcheriakov A.A., Iurev G.O., Luttsev M.D., Podolsky N.E., Ageev S.V., Petrov A.V., Vasina L.V., Solovtsova I.L., Sharoyko V.V., Murin I.V. (2020). Physicochemical properties, biological activity and biocompatibility of water-soluble C_60_-Hyp adduct. Colloids Surf. B Biointerfaces.

[14] Wu G., Gao X.J., Jang J., Gao X. (2016). Fullerenes and their derivatives as inhibitors of tumor necrosis factor-α with highly promoted affinities. J. Mol. Modeling.

[15] Bakry R., Vallant R.M., Najam-ul-Haq M., Rainer M., Szabo Z., Huck C.W., Bonn G.K. (2007). Medicinal applications of fullerenes. Int. J. Nanomed..

[16] Gaur M., Misra C., Yadav A.B., Swaroop S., Maolmhuaidh F.Ó., Bechelany M., Barhoum A. (2021). Biomedical Applications of Carbon Nanomaterials: Fullerenes, Quantum Dots, Nanotubes, Nanofibers, and Graphene. Materials.

[17] Leszek J., Md Ashraf G., Tse H.W., Zhang J., Gasiorowski K., Avila-Rodriguez F.M., Tarasov V.V., Barreto E.G., Klochkov G.S., Bachurin O.S. (2017). Nanotechnology for Alzheimer Disease. Curr. Alzheimer Res..

[18] Lahir Y. (2017). Impacts of Fullerene on Biological Systems. Clin. Immunol. Endocr. Metab. Drugs.

[19] Lin J., Zhong Z., Li Q., Tan Z., Lin T., Quan Y., Zhang D. (2017). Facile Low-Temperature Synthesis of Cellulose Nanocrystals Carrying Buckminsterfullerene and Its Radical Scavenging Property in Vitro. Biomacromolecules.

[20] Hardt J.I., Perlmutter J.S., Smith C.J., Quick K.L., Wei L., Chakraborty S.K., Dugan L.L. (2018). Pharmacokinetics and Toxicology of the Neuroprotective e,e,e-Methanofullerene(60)-63-tris Malonic Acid [C(3)] in Mice and Primates. Eur. J. Drug Metab. Pharmacokinet..

[21] Fluri F., Grünstein D., Cam E., Ungethuem U., Hatz F., Schäfer J., Samnick S., Israel I., Kleinschnitz C., Orts-Gil G. (2015). Fullerenols and glucosamine fullerenes reduce infarct volume and cerebral inflammation after ischemic stroke in normotensive and hypertensive rats. Exp. Neurol..

[22] Huang S.S., Tsai S.K., Chih C.L., Chiang L.-Y., Hsieh H.M., Teng C.M., Tsai M.C. (2001). Neuroprotective effect of hexasulfobutylated C60 on rats subjected to focal cerebral ischemia. Free. Radic. Biol. Med..

[23] Lin A.M.-Y., Fang S.-F., Lin S.-Z., Chou C.-K., Luh T.-Y., Ho L.-T. (2002). Local carboxyfullerene protects cortical infarction in rat brain. Neurosci. Res..

[24] Vani J.R., Mohammadi M.T., Foroshani M.S., Jafari M. (2016). Polyhydroxylated fullerene nanoparticles attenuate brain infarction and oxidative stress in rat model of ischemic stroke. EXCLI J..

[25] Hsieh F.-Y., Shrestha L.K., Ariga K., Hsu S.-H. (2017). Neural differentiation on aligned fullerene C60 nanowhiskers. Chem. Commun..

[26] Lee J.-R., Ryu S., Kim S., Kim B.-S. (2015). Behaviors of stem cells on carbon nanotube. Biomater. Res..

[27] Hsieh F.-Y., Zhilenkov A.V., Voronov I.I., Khakina E.A., Mischenko D.V., Troshin P.A., Hsu S.-H. (2017). Water-Soluble Fullerene Derivatives as Brain Medicine: Surface Chemistry Determines If They Are Neuroprotective and Antitumor. ACS Appl. Mater. Interfaces.

[28] Hu Z., Huang Y., Guan W., Zhang J., Wang F., Zhao L. (2010). The protective activities of water-soluble C60 derivatives against nitric oxide-induced cytotoxicity in rat pheochromocytoma cells. Biomaterials.

[29] Gilyarov A.V. (2008). Nestin in central nervous system cells. Neurosci. Behav. Physiol..

[30] Crane A.M., Bhattacharya S.K., Wright B., Connon C.J. (2013). The Use of Bromodeoxyuridine Incorporation Assays to Assess Corneal Stem Cell Proliferation. Corneal Regenerative Medicine: Methods and Protocols.

[31] Soltani M.H., Pichardo R., Song Z., Sangha N., Camacho F., Satyamoorthy K., Sangueza O.P., Setaluri V. (2005). Microtubule-associated protein 2, a marker of neuronal differentiation, induces mitotic defects, inhibits growth of melanoma cells, and predicts metastatic potential of cutaneous melanoma. Am. J. Pathol..

[32] Baba H., Nakahira K., Morita N., Tanaka F., Akita H., Ikenaka K. (1997). GFAP Gene Expression during Development of Astrocyte. Dev. Neurosci..

[33] Akter M., Kaneko N., Sawamoto K. (2021). Neurogenesis and neuronal migration in the postnatal ventricular-subventricular zone: Similarities and dissimilarities between rodents and primates. Neurosci. Res..

[34] He L., He T., Farrar S., Ji L., Liu T., Ma X. (2017). Antioxidants Maintain Cellular Redox Homeostasis by Elimination of Reactive Oxygen Species. Cell. Physiol. Biochem..

[35] Reiter R.J., Melchiorri D., Sewerynek E., Poeggeler B., Barlow-Walden L., Chuang J., Ortiz G.G., AcuñaCastroviejo D. (1995). A review of the evidence supporting melatonin's role as an antioxidant. J. Pineal Res..

[36] Ross K.E., Gray J.J., Winter N.A., Linseman A.D. (2012). Immunocal® and Preservation of Glutathione as a Novel Neuroprotective Strategy for Degenerative Disorders of the Nervous System. Recent Pat. CNS Drug Discov..

[37] Gharbi N., Pressac M., Hadchouel M., Szwarc H., Wilson S.R., Moussa F. (2005). Fullerene is a Powerful Antioxidant in Vivo with No Acute or Subacute Toxicity. Nano Lett..

[38] Sayes C.M., Fortner J.D., Guo W., Lyon D., Boyd A.M., Ausman K.D., Tao Y.J., Sitharaman B., Wilson L.J., Hughes J.B. (2004). The Differential Cytotoxicity of Water-Soluble Fullerenes. Nano Lett..

[39] Usenko C.Y., Harper S.L., Tanguay R.L. (2007). In vivo evaluation of carbon fullerene toxicity using embryonic zebrafish. Carbon.

[40] Xing L., Wilsch-Bräuninger M., Huttner W.B. (2021). How neural stem cells contribute to neocortex development. Biochem. Soc. Trans..

[41] Kahroba H., Ramezani B., Maadi H., Sadeghi M.R., Jaberie H., Ramezani F. (2021). The role of Nrf2 in neural stem/progenitors cells: From maintaining stemness and self-renewal to promoting differentiation capability and facilitating therapeutic application in neurodegenerative disease. Ageing Res. Rev..

[42] Paratcha G., Ibáñez C.F., Ledda F. (2006). GDNF is a chemoattractant factor for neuronal precursor cells in the rostral migratory stream. Mol. Cell. Neurosci..

[43] Liu X., Ren H., Peng A., Cheng H., Chen J., Xia X., Liu T., Wang X. (2022). The Effect of RADA16-I and CDNF on Neurogenesis and Neuroprotection in Brain Ischemia-Reperfusion Injury. Int. J. Mol. Sci..

[44] Tseng K.-Y., Anttila J.E., Khodosevich K., Tuominen R.K., Lindahl M., Domanskyi A., Airavaara M. (2018). MANF Promotes Differentiation and Migration of Neural Progenitor Cells with Potential Neural Regenerative Effects in Stroke. Mol. Ther..

[45] Goodarzi S., Da Ros T., Conde J., Sefat F., Mozafari M. (2017). Fullerene: Biomedical engineers get to revisit an old friend. Mater. Today.

[46] Andrade E.-B., Martínez A. (2017). Free radical scavenger properties of metal-fullerenes: C60 and C82 with Cu, Ag and Au (atoms and tetramers). Comput. Theor. Chem..

[47] Sohn S.J., Yu J.M., Lee E.Y., Nam Y.J., Kim J., Kang S., Kim D.H., Kim A., Kang S. (2018). Anti-aging Properties of Conditioned Media of Epidermal Progenitor Cells Derived from Mesenchymal Stem Cells. Dermatol. Ther..

[48] Konyalioglu S., Armagan G., Yalcin A., Atalayin C., Dagci T. (2013). Effects of resveratrol on hydrogen peroxide-induced oxidative stress in embryonic neural stem cells. Neural. Regen. Res..

[49] Abdanipour A., Jafari Anarkooli I., Shokri S., Ghorbanlou M., Bayati V., Nejatbakhsh R. (2018). Neuroprotective effects of selegiline on rat neural stem cells treated with hydrogen peroxide. Biomed. Rep..

[50] Hachem L.D., Mothe A.J., Tator C.H. (2015). Effect of BDNF and Other Potential Survival Factors in Models of In Vitro Oxidative Stress on Adult Spinal Cord–Derived Neural Stem/Progenitor Cells. BioRes. Open Access.

[51] Li Q., Wang P., Huang C., Chen B., Liu J., Zhao M., Zhao J. (2019). N-Acetyl Serotonin Protects Neural Progenitor Cells Against Oxidative Stress-Induced Apoptosis and Improves Neurogenesis in Adult Mouse Hippocampus Following Traumatic Brain Injury. J. Mol. Neurosci..

[52] Wang S., Huang L., Zhang Y., Peng Y., Wang X., Peng Y. (2018). Protective Effects of L-3-n-Butylphthalide Against H_2_O_2_-Induced Injury in Neural Stem Cells by Activation of PI3K/Akt and Mash1 Pathway. Neuroscience.

[53] Sun T., Wang X.-J., Xie S.-S., Zhang D.-L., Wang X.-P., Li B.-Q., Ma W., Xin H. (2011). A comparison of proliferative capacity and passaging potential between neural stem and progenitor cells in adherent and neurosphere cultures. Int. J. Dev. Neurosci..

