# Supplementary material for: Water-Soluble, Alanine-Modified Fullerene C60 Promotes the Proliferation and Neuronal Differentiation of Neural Stem Cells
Source: Int J Mol Sci. 2022 May 20;23(10):5714. doi: 10.3390/ijms23105714 (PMC9146176; doi:10.3390/ijms23105714)
Supplement: Supplementary file 1 [file ijms-23-05714-s001.zip › supplementary figures.pdf]

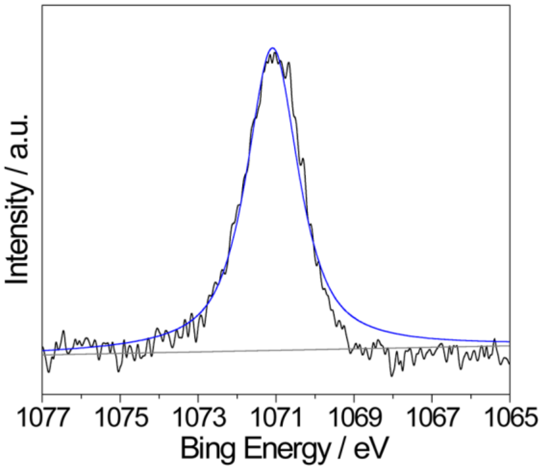

Figure S1. High-resolution XPS spectra of Na1s of Ala-C_{60}.

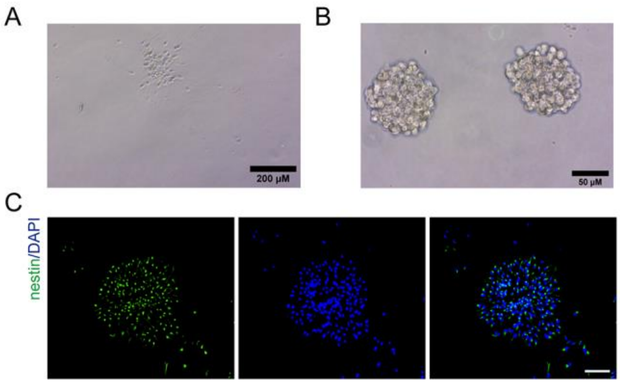

Figure S2. (a) Optical microscopy images of the adherent morphology of NSCs. Scale bar = 200 μm. (b) Optical microscopy images of the suspend neurospheres. Scale bar = 50 μm. (C) Nestin-positive cells (green) were identified via immunohistochemical staining. DAPI (blue) was used to label the nuclei. Scale bar = 100 μm.

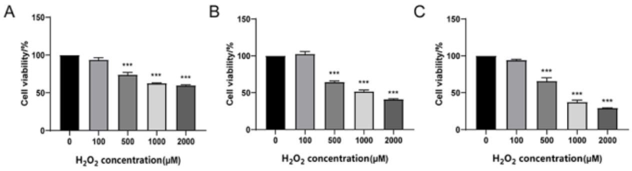

Figure S3. Cell viability of cultured NSCs treated with H_2O_2 for 24 h (a), cultured NSCs treated with H_2O_2 for 48 h (b) and cultured NSCs treated with H_2O_2 for 72 h (C). Values represent the mean±SEM, * $p < 0.05$ indicate statistical significance between H_2O_2 treatment group and H_2O_2 0 μM group. All statistical according to ANOVA followed by LSD post hoc analysis.
