# Supplementary material for: Water-Soluble, Alanine-Modified Fullerene C60 Promotes the Proliferation and Neuronal Differentiation of Neural Stem Cells
Source: Int J Mol Sci. 2022 May 20;23(10):5714. doi: 10.3390/ijms23105714 (PMC9146176; doi:10.3390/ijms23105714)
Supplement: Supplementary file 1 [file ijms-23-05714-s001.zip › suppplementalry information.pdf]

Calculation of the formula of the *β*-Ala-C_{60}

Based on the results from XPS, the content of C, O, N and Na were determined to be 73.3%, 23.15%, 2.09% and 2.46% (by atom), respectively. According to the weight ratio C/Na and C/O, the following two equations can be obtained:

C/O:

$$(60+3a+16d)/(2a+m+n) = 73.3/22.15 \quad (1)$$

C/N:

$$(60+3a+16d)/(a+d) = 73.3/2.09 \quad (2)$$

C/Na:

$$720/23y=(60+3a+16d)/c = 73.3/2.46 \quad (3)$$

By the law of charge conservation: (b, c, d dissociate from a, m)

$$a+m = b+c+d \quad (4)$$

From TGA, the content of secondary water is 11.43 wt%. Based on this result, equation 5 can be obtained:

$$\{12\times(60+3a+16d)+14(a+d)+16(2a+m+n)+23c+(6a+b+32d+2n)\} \times 11.43\% = 18n \quad (5)$$

Based on the C, H ratio of 55.93% to 3.505% determined by elemental analysis:

$$12(60+3a+16d)/(6a+b+32d+2n) = 50.93\%/3.505\% \quad (6)$$

According to equations 1-6, a=2.10, m=9.05, b=8.33, c=2.43, d=0.382, n=8.64, and the formula of the β-Ala-C_{60} can be calculated to be $C_{60}(NH_2\text{-}CH(CH_3)\text{-}COO^-)_{2.1}O_{9.05}^-H_{8.33}Na_{2.43}TBA_{0.382}\cdot 8.64H_2O$, which is approximately to $C_{60}(NH\text{-}CH(CH_3)\text{-}COO^-)_2O^-_9H^+_8Na^+_2TBA^+\cdot 9H_2O$.
